# Porous Poly(4-vinylpyridine) Coatings Prepared by Electrostatic Slurry Spray Deposition: Morphological Control and Preliminary Formaldehyde-Reduction Assessment

**DOI:** 10.3390/ma19214744

**Published:** 2026-11-08

**Authors:** Sangho Park, Jungmin Kim, Sewook Lee, Dongwook Shin

**Affiliations:** Division of Materials Science & Engineering, Hanyang University, Seongdong-gu, Seoul 04763, Republic of Korea; clickpsh@hanyang.ac.kr (S.P.); ohyes7017@hanyang.ac.kr (J.K.); lee01ok@gmail.com (S.L.)

**Keywords:** poly(4-vinylpyridine), formaldehyde removal, slurry spray deposition, microstructure control

## Abstract

**Highlights:**

• Electrostatic slurry spray deposition enabled porous P4VP coating fabrication.
• An ethanol/water composite slurry provided controlled P4VP precipitation.
• Composite-slurry processing produced porous particulate coatings; hollow-like morphologies are discussed as a possible interpretation.
• During a preliminary 30 m^3^ chamber test, the HCHO concentration decreased by 0.144 ppm in 1 h.
• An apparent concentration-decay-based air-cleaning rate (Q_app_) of approximately 5.69 m^3^ h^−1^ was calculated without background correction.

**Abstract:**

Porous poly(4-vinylpyridine) (P4VP) coatings were fabricated by electrostatic slurry spray deposition (ESSD) using solution and ethanol–water composite-slurry precursors. Thirty-four solvents were screened to examine the relationship between precursor formulation, spray behavior, and deposited morphology. Gradual nonsolvent addition generated a particulate composite slurry, with a principal volume-weighted dynamic light scattering (DLS) peak at 13.78 nm for the 7.4 wt.% formulation. Under the investigated conditions, this slurry produced a more spatially uniform porous coating than the solution precursor and was successfully deposited onto a three-dimensional fibrous filter scaffold. During a preliminary 30 m^3^ chamber assessment of the air-purifier system equipped with the coated filter, formaldehyde (HCHO) concentration decreased from 0.846 to 0.702 ppm in 1 h, corresponding to an apparent concentration-decay-based air-cleaning rate (Q_app_) of approximately 5.69 m^3^ h^−1^. This preliminary observation describes the performance of the assembled air-purifier system; the principal contribution of the study is the formulation and morphological control of porous P4VP coatings through composite-slurry ESSD.

## 1. Introduction

Formaldehyde (HCHO) is an important industrial chemical that is widely used in the production of resins, polymers, and other commercial products [1,2]. Despite its industrial utility, formaldehyde exposure has been associated with a range of acute and chronic adverse health effects [3,4]. Reproductive and developmental effects associated with formaldehyde exposure have also been systematically reviewed [5]. The World Health Organization (WHO) has established an indoor-air guideline for formaldehyde based on short-term exposure [6]. In addition, the Centers for Disease Control and Prevention (CDC) has provided guidance concerning formaldehyde exposure in residential environments [7]. Consequently, formaldehyde control is routinely incorporated into commercial air-cleaning devices. Various approaches have been investigated for the removal of volatile organic compounds (VOCs) from indoor air. Photo-catalytic air-cleaning technologies have been studied extensively for VOC degradation, although their effectiveness depends on the target compounds and operating conditions [8]. Ozone-based treatment in combination with porous materials has also been investigated for the removal of indoor VOCs [9]. Portable room air cleaners using sorption-based media have been evaluated for controlling VOC concentrations in indoor environments [10], while biological treatment has been investigated as an alternative approach for indoor VOC removal [11]. UV-assisted photocatalytic oxidation (UV-PCO) has also been evaluated for the removal of multiple VOCs [12], and the capabilities and limitations of photocatalytic purification for indoor-air treatment have been reviewed extensively [13]. In addition, activated-carbon filtration has been investigated in relation to VOC loading and ozone-removal performance [14]. Despite the availability of these air-treatment technologies, the development of simple and effective materials for the removal of gaseous formaldehyde remains important. In particular, filtration materials that provide a large accessible surface area together with chemical affinity toward HCHO are desirable for efficient formaldehyde capture. Polymeric materials have been widely investigated for functional filtration and sensing applications because of their processability and structural versatility. Electrospun polymer nanofibers, for example, provide highly accessible fibrous architectures for various functional applications [15]. Polymeric nanostructures have also been employed as chemical-sensing platforms [16], while conducting polymers have been investigated for gas-sensing applications [17]. Amine-functionalized polymer films have been investigated for gaseous formaldehyde detection through chemical interactions with formaldehyde [18]. P4VP contains pyridine nitrogen functionalities that can provide interaction sites for polar molecules. In this study, P4VP was therefore investigated as a functional polymer for HCHO removal, with particular emphasis on increasing gas–polymer contact through control of the porous membrane structure. For effective gas filtration, a membrane structure that provides extensive contact between the functional material and target gas molecules is desirable. Nanofibrous structures with highly accessible surface areas have therefore been investigated for sensitive formaldehyde detection [19]. From this perspective, controlling the precipitation behavior of P4VP and the resulting porous membrane morphology is important for enhancing contact between P4VP and gaseous formaldehyde. Electrostatic slurry spray deposition (ESSD) has been employed to fabricate continuously graded anode functional layers for proton-conducting fuel cells [20]. The technique has also been applied to continuously graded composite cathodes, demonstrating its applicability to the controlled deposition of functional particulate layers [21]. These studies indicate that ESSD provides a versatile route for controlling the structure of deposited functional materials. However, the formation of well-defined porous P4VP membranes strongly depends on precursor solubility and polymer precipitation kinetics during electrostatic spraying. Nevertheless, the relationship between precursor state, precipitation behavior, and porous structure formation in P4VP membranes remains poorly understood.

Nitrogen-containing surface groups and pore architecture have been reported to influence formaldehyde adsorption in nanoporous carbon adsorbents [22]. A separate study of resin-based carbons likewise identified important roles for pore structure and nitrogen surface groups in formaldehyde adsorption [23]. These findings highlight the potential benefits of combining accessible porous structures with nitrogen-containing functionalities for HCHO capture. Molecular rotational spectroscopy has identified an n→π* interaction in a pyridine–formaldehyde adduct [24], providing a molecular-level rationale for investigating potential interactions between the pyridine groups of P4VP and formaldehyde. Porous P4VP has also been studied as a host for electron-accepting molecules by in situ Fourier Transform Infrared Spectroscopy (FTIR) [25], further supporting interest in its pyridine functionality. Together, these studies provide a scientific basis for exploring porous P4VP coatings as candidates for formaldehyde-reduction applications.

In this study, ESSD was investigated as a route to porous P4VP coatings using ethanol–water composite slurries prepared through nonsolvent-induced precipitation. The effects of precursor state and formulation on spray behavior, particulate characteristics, and coating morphology were examined, with particular attention to improving the radial uniformity of deposition on silicon substrates. The selected slurry formulation was further applied to a three-dimensional fibrous filter scaffold. A preliminary HCHO chamber assessment was included to explore the application of the coated filter in an air-purifier system. The primary focus remains the development of a processable particulate precursor and the resulting porous-coating architecture.

## 2. Experimental Section

### 2.1. Solvent Selection

The 34 solvents summarized in Table 1 were screened by comparing their physicochemical properties with their spray behavior, with particular attention to dielectric constant, density, and viscosity. P4VP solubility was evaluated at a polymer loading of 10 wt.% relative to the solvent. Literature property sources are dielectric constant [26], density and viscosity [27], and surface tension [28], with solvent-specific exceptions [29,30,31]. Literature values are primarily at 25 °C; † indicates 20 °C where specified. P4VP solubility and spray stability were evaluated experimentally rather than obtained from literature. The solvents used for experimental screening were obtained from four commercial suppliers: Samchun Pure Chemical Co., Ltd. (Pyeongtaek, Republic of Korea), Sigma-Aldrich (St. Louis, MO, USA), Daejung Chemicals & Metals Co., Ltd. (Siheung, Republic of Korea), and Junsei Chemical Co., Ltd. (Tokyo, Japan).

Poly(4-vinylpyridine) (P4VP; Sigma-Aldrich, St. Louis, MO, USA; reported reported Mw ≈ 60,000) was investigated in solution and composite-slurry precursor forms. Ethanol (99.9%; Daejung Chemicals & Metals Co., Ltd., Siheung, Republic of Korea) and deionized water (18.2 MΩ·cm; ELGA PURELAB Option, ELGA LabWater, High Wycombe, UK) were used. Solution-type and composite-slurry precursors were distinguished by the state of the polymer, and spray stability was assessed by maintenance of a steady cone-jet for more than 1 h.

### 2.2. Formulation of P4VP Solution and Slurry Precursors

The solution precursor was prepared by dissolving 0.1 g of P4VP in 10 mL of ethanol. Preliminary spraying experiments were conducted with selected pure solvents without P4VP. The spray-footprint diameters summarized in Table 2 represent the lateral extent of the observed spray footprint under the specified conditions, rather than the diameter of a uniformly coated area.

To obtain a sprayable particulate precursor, P4VP was first dissolved in ethanol, followed by gradual addition of deionized water as a nonsolvent. Controlled addition was used to promote fine P4VP-containing precipitates while limiting abrupt formation of coarse aggregates. The resulting composite slurry was evaluated for stable ESSD and porous particulate coating formation.

For composite-slurry preparation, P4VP was dissolved in 28 mL of ethanol, and 28 mL of deionized water was subsequently added at 0.5 mL min^−1^ in 1 mL increments separated by 5 min intervals under stirring. After a further 10 min of mixing, the slurry was used without prolonged storage. P4VP concentrations in Table 3 were calculated from the total masses of polymer and solvents (ethanol:water = 1:1, *v*/*v*; densities of 0.789 and 1.000 g mL^−1^, respectively). The 7.4 wt.% formulation contained 4.0 g of P4VP. Dynamic viscosity was determined using an Ubbelohde capillary viscometer (SI Analytics GmbH, Mainz, Germany) from measured efflux times and composition-estimated slurry density (η = ρν); three measurements were performed per formulation. The measured slurry viscosity reported in Table 4 was used as the viscosity input for DLS size calculation. DLS measurements were performed directly on undiluted slurry using a Malvern Zetasizer Nano (Malvern Panalytical, Malvern, UK) equipped with Zetasizer Software (version 7.11) under ambient laboratory conditions without active temperature or humidity control. The refractive index was specified on the basis of the ethanol–water carrier composition (1:1, *v*/*v*). For FE-SEM analysis, particulate material was recovered by vacuum filtration for approximately 24 h using a 20 nm pore-size alumina upper membrane (Whatman Anodisc 13, Cat. No. 6809-7003; GE Healthcare Life Sciences, Little Chalfont, UK) supported by a 0.45 μm pore-size PTFE lower membrane (Cat. No. SL.HP045047D; supplied by SciLab Korea Co., Ltd., Seoul, Republic of Korea); the retained material was dried at 30 °C and Au-coated before imaging. A density-based conversion using literature component properties [32] was applied to the Ubbelohde measurements.

### 2.3. ESSD Technique and Fabrication of the Filter Membrane

**Figure 1:**
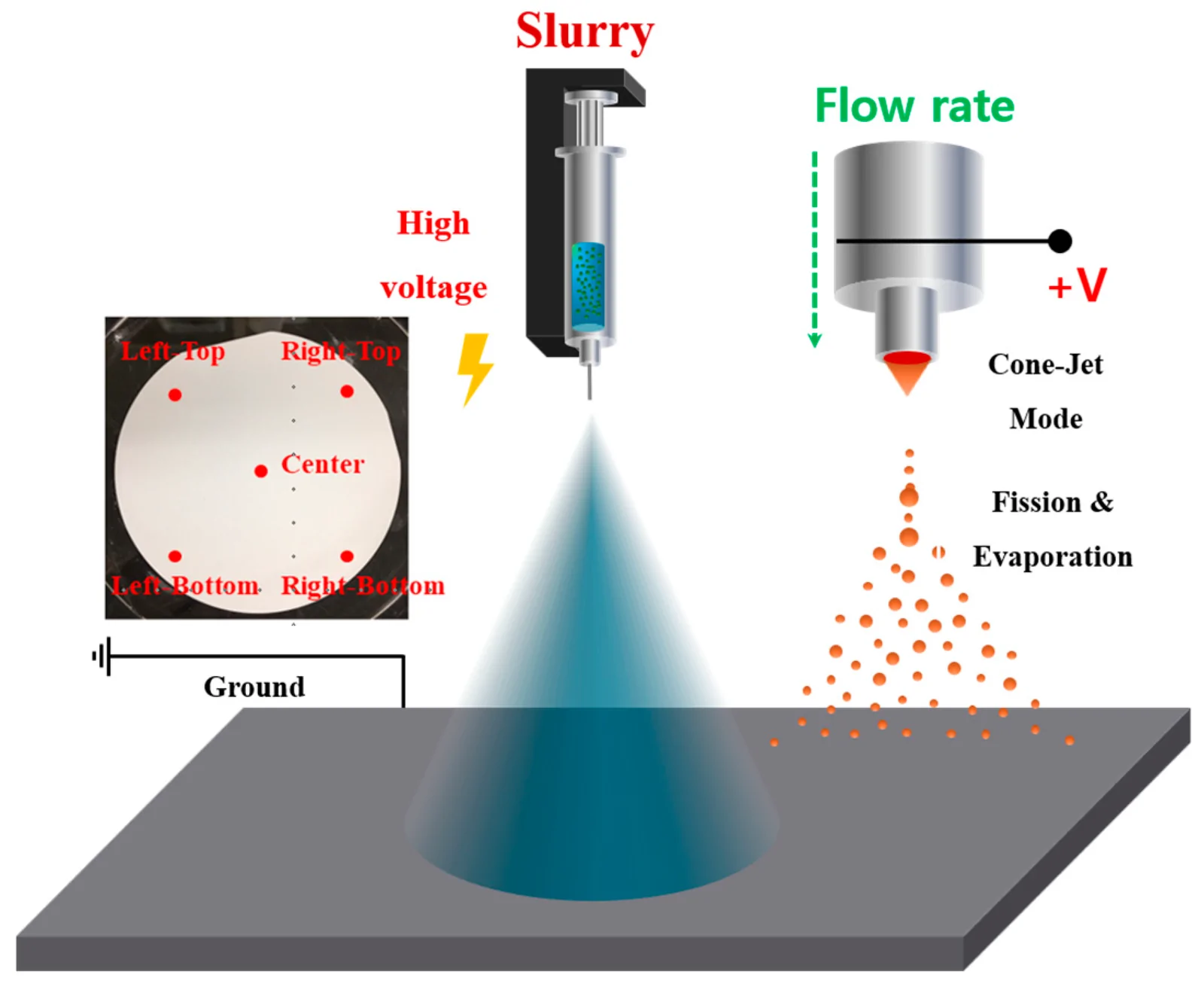
Schematic illustration of the electrostatic slurry spray deposition (ESSD) technique used to fabricate supported P4VP coatings.

Figure 1 depicts the ESSD apparatus, including a syringe pump (supplied by Sercrim Labtech, Seoul, Republic of Korea), high-voltage DC supply (supplied by KSC Korea Switching, Seoul, Republic of Korea), nozzle (NanoNC, Seoul, Republic of Korea), and grounded substrate. Silicon-wafer deposition used +10 kV, a 12 cm nozzle-to-substrate distance, a 3 mL h^−1^ flow rate, and a nozzle inner diameter of 3 mm. Deposition onto the fibrous filter scaffold used +13 kV, 20 cm, and 5 mL h^−1^ for ap-proximately 1 h. Experiments were conducted at approximately 25 °C and 40% relative humidity without active environmental control.

Coating surfaces were characterized using FE-SEM (S-4300SE; Hitachi High-Technologies Corporation, Tokyo, Japan) and a 3D optical profiler (Vision ZIP250; Optical Gaging Products, Rochester, NY, USA). Before FE-SEM, samples were Au sputter-coated using a Cressington Sputter Coater 108 (Cressington Scientific Instruments Ltd., Watford, UK; 20 mA, 30 s). FE-SEM accelerating voltages were 15 kV for solution-derived coatings, recovered particulate material, coating cross sections, and coatings examined during the 8 h spraying experiment; 20 kV for coatings prepared at different slurry concentrations; and 5 kV for the fibrous filter scaffold. For the 7.4 wt.% composite slurry, samples collected during an 8 h spraying experiment were examined, and viscosity was measured before and after spraying. Coating thickness was observed at the wafer center and four peripheral positions.

### 2.4. Formaldehyde (HCHO) Filter Performance

FTIR spectroscopy (IRAffinity-1; Shimadzu Corporation, Kyoto, Japan) was used to examine the retention of characteristic P4VP functional groups following solution and composite-slurry processing and ESSD deposition. Spectra of untreated P4VP and material recovered after solution- and slurry-based deposition were compared. For spectral comparison, each FTIR spectrum was normalized to its own maximum peak intensity.

A preliminary HCHO concentration-decay assessment was performed by an external home-appliance manufacturer using an air purifier equipped with a P4VP-coated fibrous filter scaffold (approximately 32 × 38 cm). The manufacturer reported a nominal chamber volume of 30 m^3^. HCHO was introduced toward a target concentration of approximately 1 ppm, and the supply was stopped before the air purifier was operated at its highest fan setting for 60 min. The initial measured concentration was 0.846 ppm, and the resulting concentration–time data were used for the analysis described below. The test was conducted at ambient temperature and atmospheric pressure as reported by the evaluator.

## 3. Results and Discussion

Spray stability for the 34 screened solvents was assessed by the ability to sustain a steady cone-jet for more than 1 h. Spray behavior depends jointly on precursor formulation, solvent properties, and operating conditions, and the solvent-screening results provide a basis for selecting formulations suitable for stable ESSD operation.

Tang and Gomez [33] demonstrated the influence of liquid electrical properties on cone-jet electrospraying, while Gañán-Calvo and co-workers [34] reported scaling relationships involving spray current and droplet size. Together, these studies provide a theoretical basis for interpreting the observed spray behavior in terms of precursor properties, including viscosity, surface tension, and electrical characteristics, as well as the applied operating conditions.

**Figure 2:**
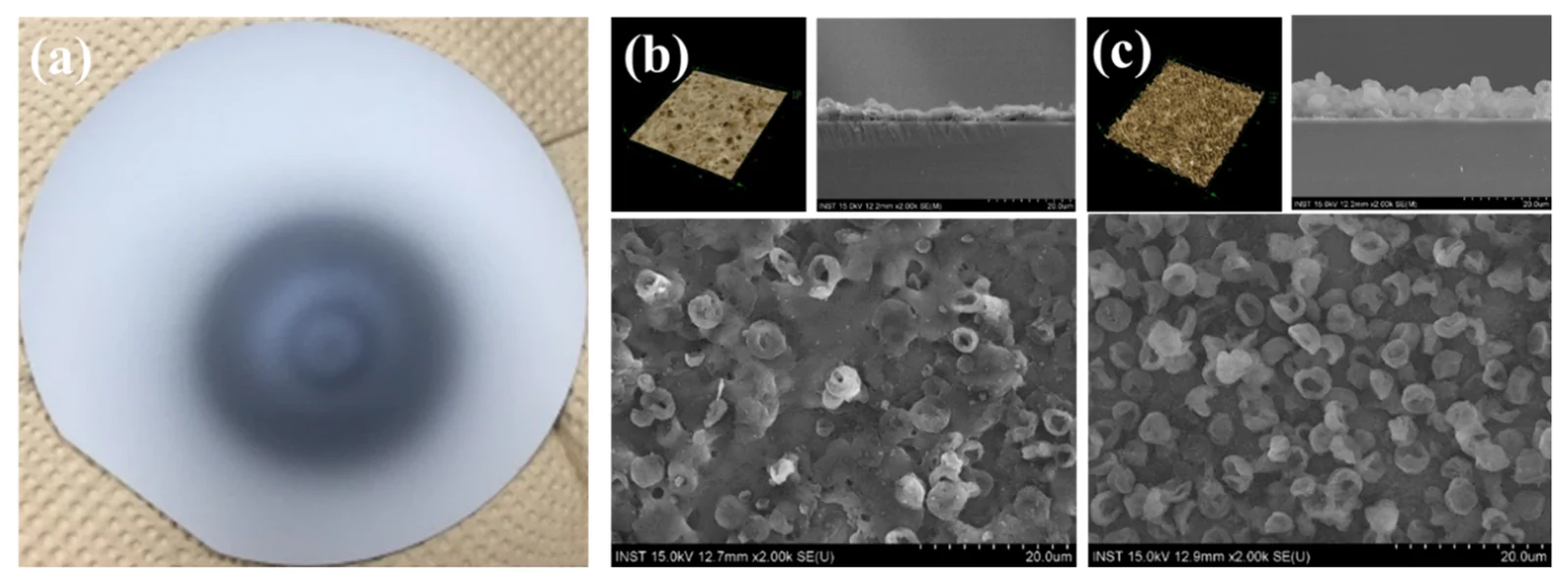
Morphological characterization of the P4VP coating prepared from the solution precursor by ESSD: (**a**) macroscopic appearance; (**b**) central deposition region; and (**c**) peripheral deposition region. Panels (**b**,**c**) each show a 3D surface profile (**upper left**), cross-sectional FE-SEM image (**upper right**), and surface FE-SEM image (**bottom**).

Under the investigated conditions, the ethanol-based solution precursor produced the radially non-uniform deposit shown in Figure 2a, with a visibly denser central region and more particulate peripheral region. Figure 2b represents the central region and Figure 2c the peripheral region; each includes a 3D surface profile, cross-sectional FE-SEM image, and surface FE-SEM image.

The peripheral deposit appeared thicker and more porous than the central region and exhibited concave or shell-like particle features. Radial differences in droplet flux, solvent evaporation, and local deposition behavior may contribute to this morphology. Similar exterior particle features have been discussed in studies of droplet drying and polymer electrospraying [35,36,37], providing a qualitative basis for considering evaporation-driven material redistribution during ESSD.

To improve the radial uniformity observed with the solution precursor, ethanol was combined with deionized water to form a composite-slurry precursor through nonsolvent-induced precipitation (Table 3). This approach was examined as an alternative deposition route, with emphasis on the spatial distribution and particulate morphology of the resulting coating.

**Figure 3:**
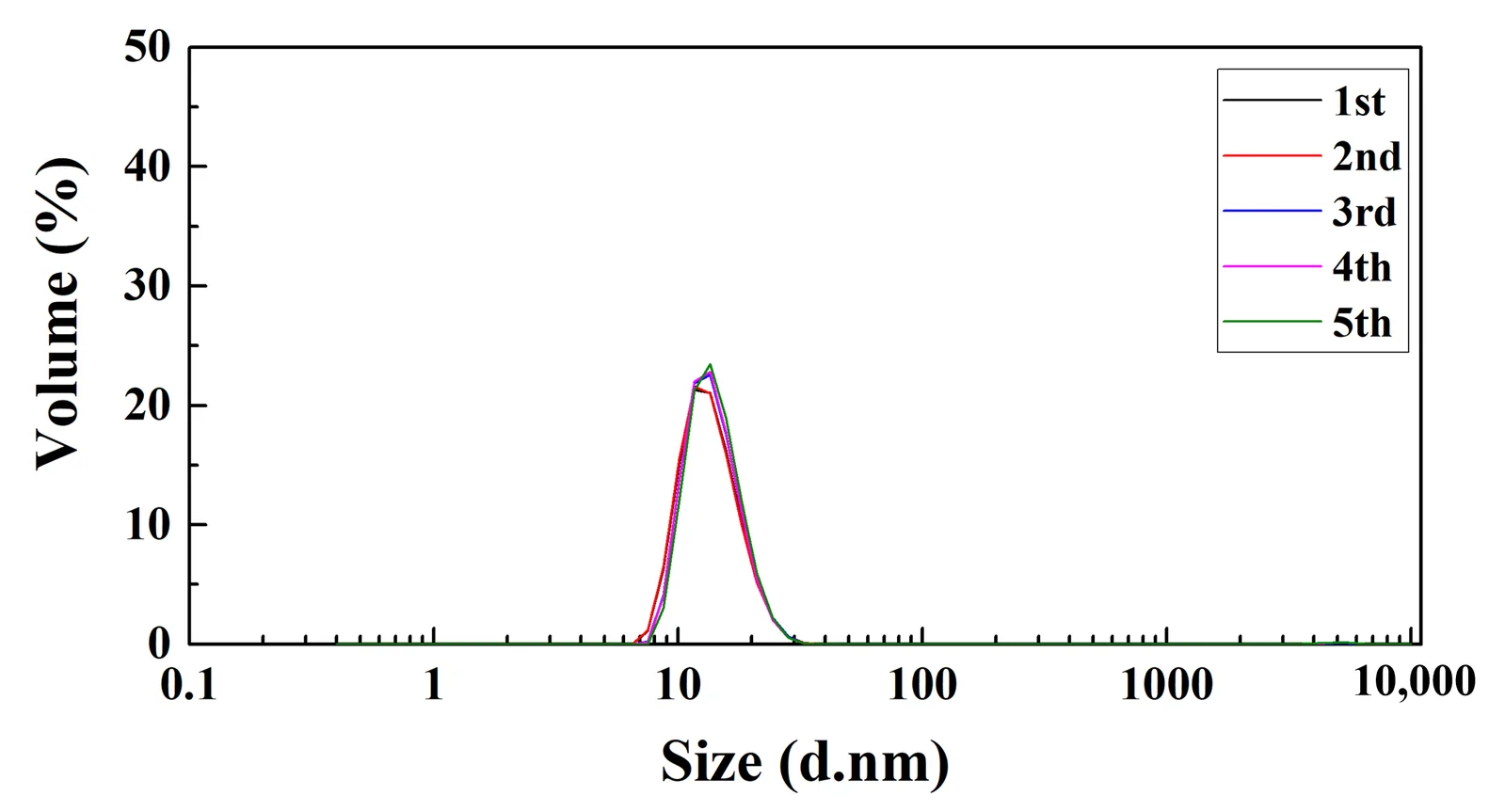
Volume-weighted DLS particle-size distributions obtained from five aliquots of the 7.4 wt.% P4VP composite slurry.

Figure 3 shows the volume-weighted DLS distributions obtained from five aliquots of the same 7.4 wt.% composite-slurry preparation. The principal peak was 13.78 nm, with a Z-average of 42.86 nm and a polydispersity index (PDI) of 0.285. A minor larger-size feature appeared at approximately 5263 nm (0.2% of the model-derived volume-weighted distribution), potentially reflecting a small population of aggregates or larger precipitated species. Principal peaks of 15.17 and 18.74 nm were observed for separately formulated 8.2 and 9.1 wt.% slurries, respectively, indicating a concentration-dependent change in the apparent particle-size distribution. Since the reported volume-weighted distributions are derived from DLS scattering data through a conversion model, the peak positions and relative fractions are interpreted as apparent hydrodynamic-size characteristics, rather than a direct measure of the absolute sizes and proportions of all solid particles. The DLS results are used primarily to characterize fine dispersed species and their formulation-dependent behavior.

**Figure 4:**
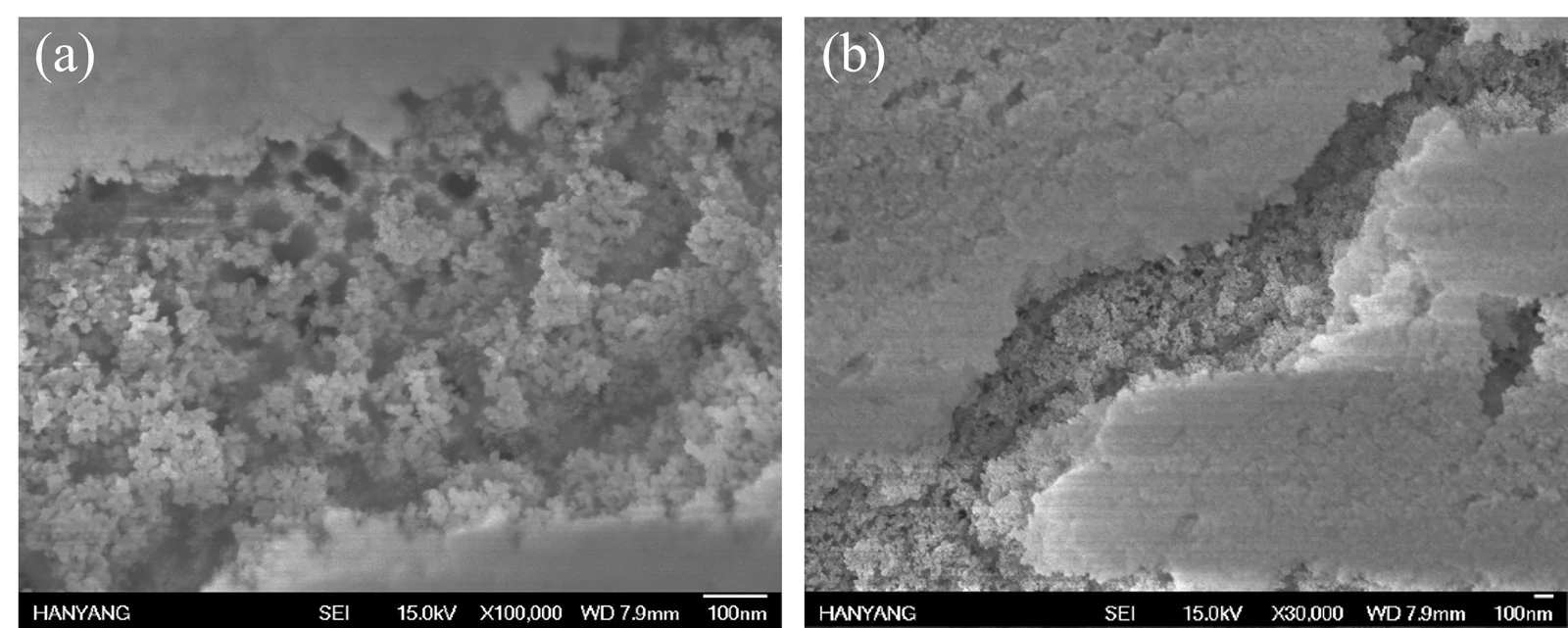
FE-SEM images of particulate material recovered from the 7.4 wt.% P4VP composite slurry by membrane filtration: (**a**) ×100,000 and (**b**) ×30,000.

Particulate material recovered from the composite slurry was examined by FE-SEM after vacuum filtration through a 20 nm pore-size alumina membrane supported by a 0.45 μm pore-size PTFE membrane, followed by drying at 30 °C and Au coating. As shown in Figure 4, the recovered material contained fine particulate features of approximately 10 nm. Together with the DLS measurements of the liquid precursor, these observations support the presence of dispersed, P4VP-containing particulate material formed during controlled nonsolvent addition. Because the FE-SEM specimens were recovered and dried, their observed dimensions are interpreted as complementary morphological evidence rather than direct liquid-state particle-size measurements.

**Figure 5:**
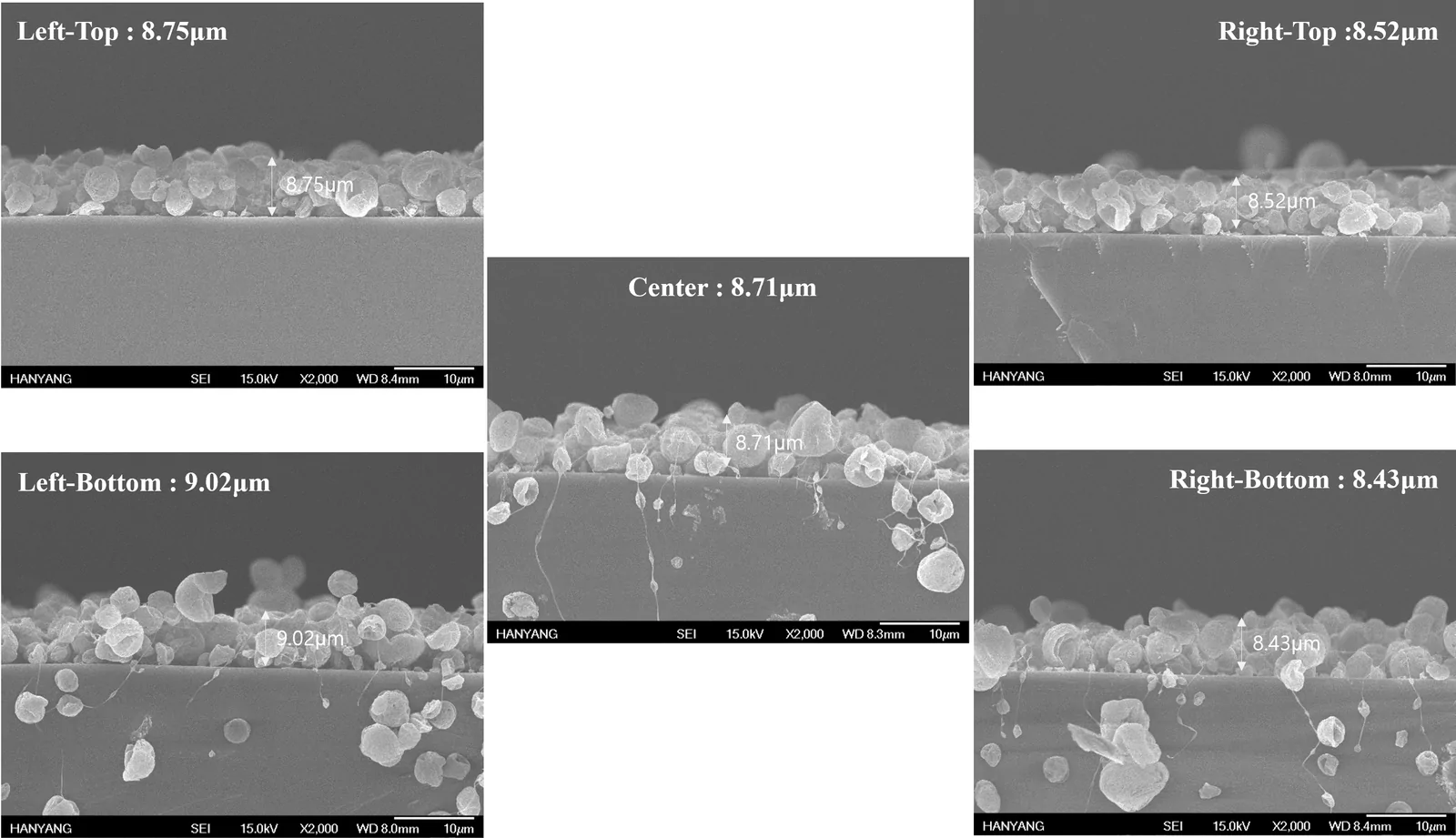
Cross-sectional FE-SEM comparison of the center and four peripheral locations in a P4VP coating deposited from the 7.4 wt.% composite slurry.

Figure 5 compares the coating cross sections at the wafer center and four peripheral positions after 10 min deposition from the 7.4 wt.% composite slurry. The observed coating was approximately 8–9 μm thick at the sampled positions and appeared more spatially consistent than the solution-derived deposit.

**Figure 6:**
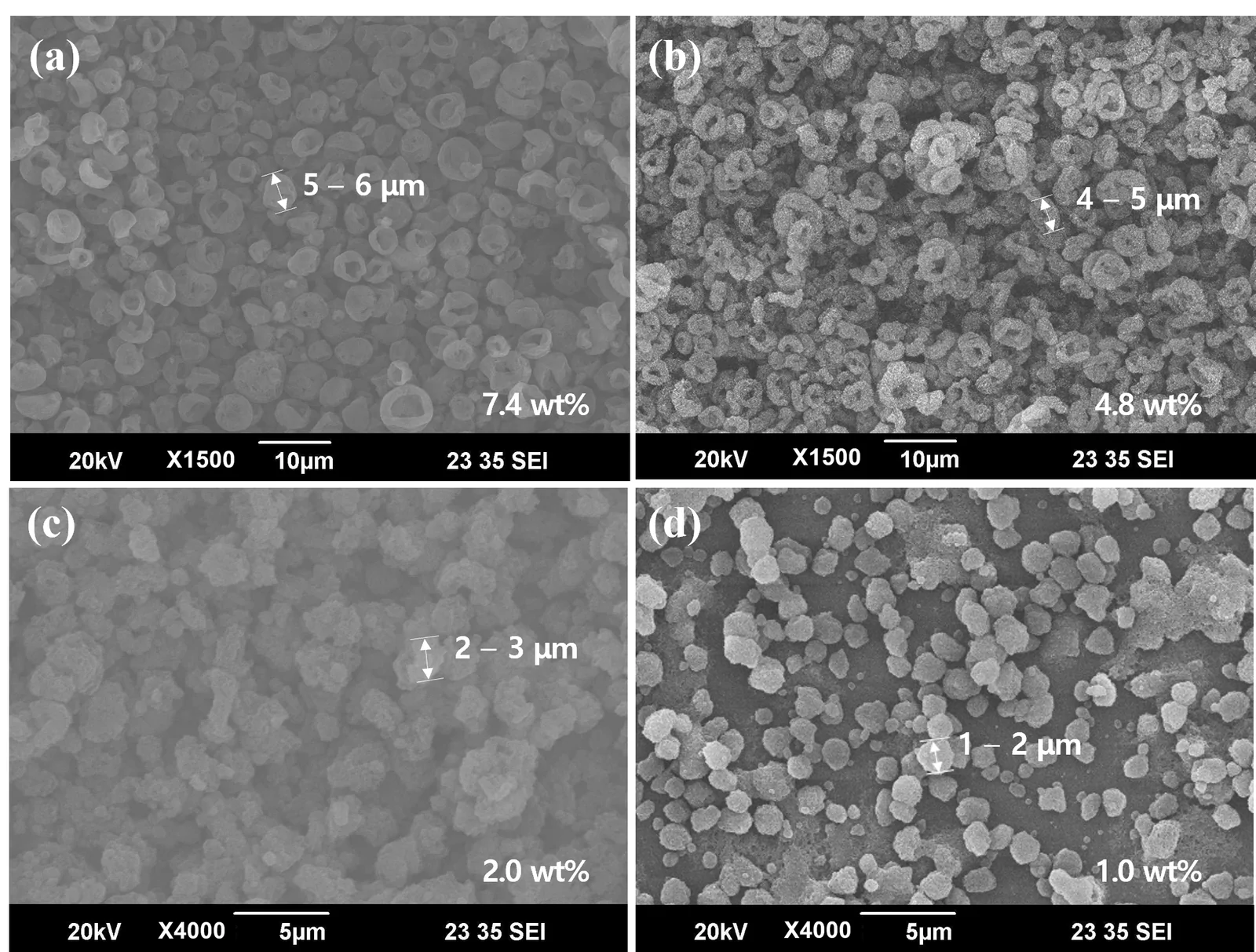
FE-SEM surface images of P4VP coatings prepared from composite slurries containing (**a**) 7.4 wt.%, (**b**) 4.8 wt.%, (**c**) 2.0 wt.%, and (**d**) 1.0 wt.% P4VP.

Figure 6 compares the deposited morphologies of composite slurries containing 7.4, 4.8, 2.0, and 1.0 wt.% P4VP. The apparent size and shape of the particulate features varied with precursor concentration, and some deposits exhibited concave or shell-like exterior morphologies. Additional deposition trials at higher concentrations showed a mixture of particulate, bead-like, and fiber-like features at 8.2 wt.%, with a more pronounced fibrous appearance at 9.1 wt.%. Together with the viscosity trend in Table 4, these observations favored selection of the 7.4 wt.% slurry for a porous particulate coating rather than a predominantly fibrous deposit.

**Figure 7:**
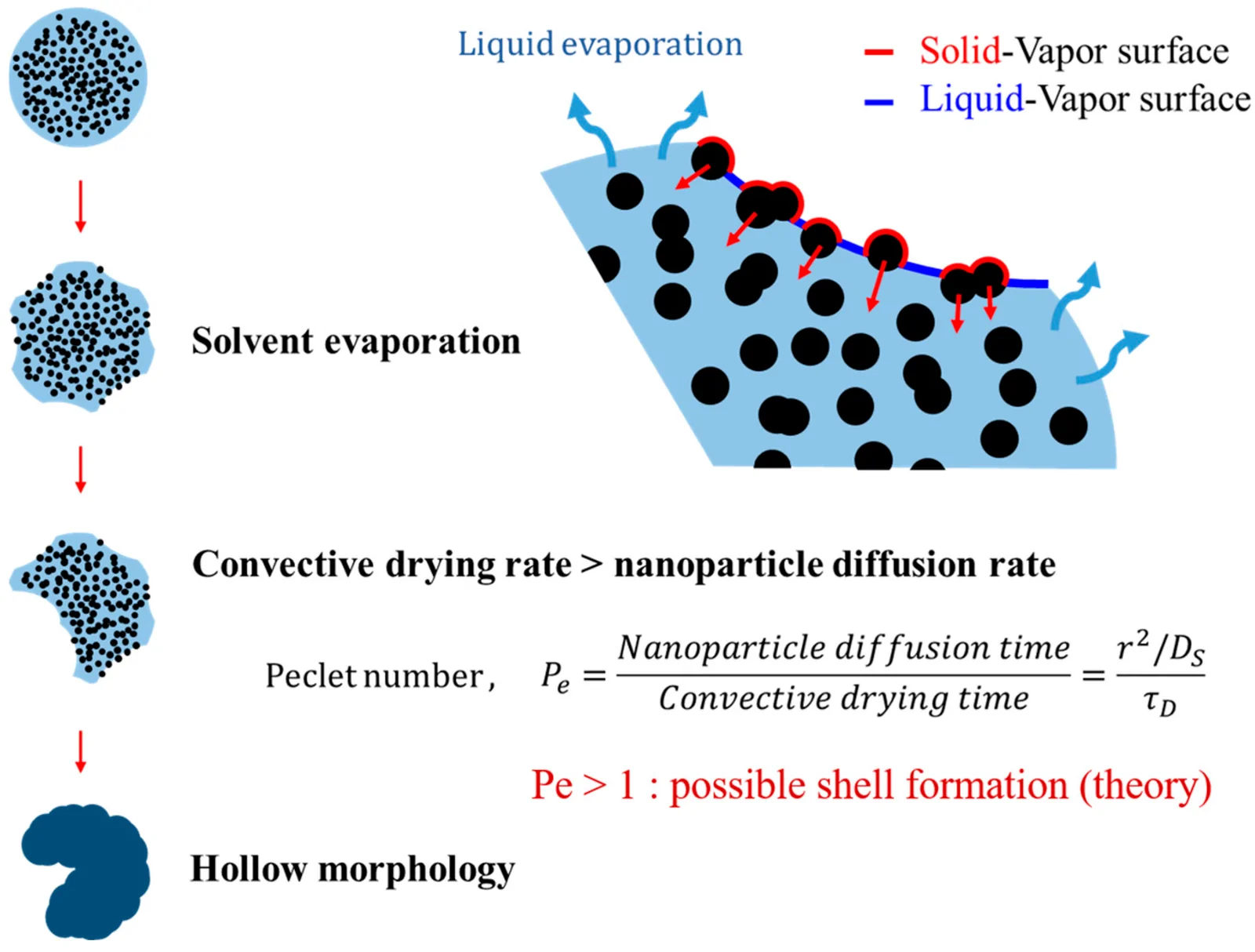
Conceptual schematic of possible P4VP particle-morphology evolution during ESSD, illustrating the competition between droplet evaporation and internal material diffusion through the Péclet-number framework [35,36,37].

Figure 7 illustrates a possible pathway for the evolution of concave or shell-like particle morphologies during ESSD. As solvent evaporates from an electrified droplet, the competition between the rate of evaporation and the diffusion of polymer-containing species may influence their redistribution toward the droplet surface. Related evaporation-driven morphology changes have been examined in silica suspension spray drying [35] and polymer electrospraying [36,37]. Although the droplet-generation processes differ, these studies provide a useful physical framework for interpreting the exterior features observed here.

In Equation (1), *Pe* is the Péclet number, *r* is the characteristic droplet radius, *D_s_* is the diffusion coefficient of dispersed species, and τ*_D_* is the characteristic droplet-drying time. A relatively high *Pe* (*Pe* > 1) indicates that surface enrichment during evaporation can outpace internal diffusion, potentially favoring shell-like morphological development [35,36,37]. The schematic is intended as a qualitative interpretation, because the quantities required to determine *Pe* for the present P4VP droplets were not measured.

(1) $$\mathrm{Pe}=\frac{r^{2}}{\tau_{D}D_{s}}$$

The dense central deposit observed for the solution precursor may be associated with local differences in droplet flux, solvent evaporation, and post-impact coalescence across the spray footprint. These factors provide a possible explanation for the radial morphological differences observed in the solution-derived coating.

The more spatially consistent porous coatings obtained from the composite slurry suggest that precursor-state control is an effective strategy for modifying ESSD deposition behavior. Nonsolvent-induced precipitation provides dispersed particulate material before spraying, while subsequent droplet evaporation and deposition can contribute to particle accumulation and interconnection on the substrate. These combined processes offer a plausible explanation for the differences between solution-derived and composite-slurry-derived coatings.

The concentration-dependent morphologies in Figure 6 are consistent with the influence of solvent evaporation and material redistribution discussed in the droplet-drying and electrospraying literature [35,36,37]. The concave and shell-like features observed by FE-SEM describe the external particle morphology; their internal structure would require further direct characterization.

**Figure 8:**
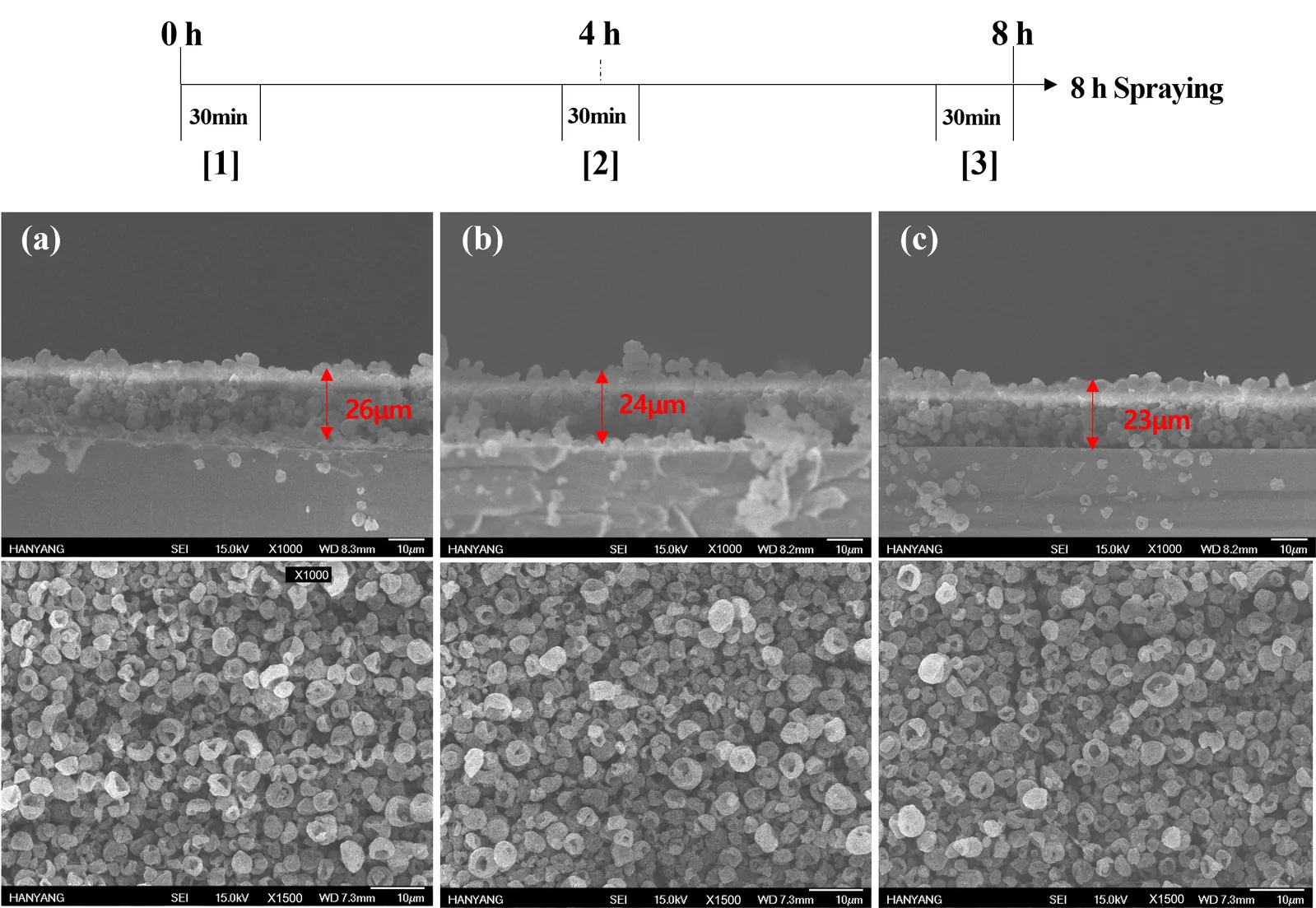
FE-SEM cross-sectional and surface images of P4VP coatings collected at (**a**) 0 h, (**b**) 4 h, and (**c**) 8 h during continuous ESSD operation.

The initial solution precursor produced pronounced radial differences in coating morphology, whereas the 7.4 wt.% composite slurry yielded a relatively uniform porous particulate layer under the investigated conditions. This formulation was therefore selected for the extended spraying experiment. Figure 8 compares coatings produced in separate 30 min deposition intervals at the beginning (0 h), middle (4 h), and end (8 h) of continuous ESSD operation.

A stable cone-jet was maintained throughout the 8 h ESSD run. The coating thicknesses measured from separately deposited 30 min specimens at 0, 4, and 8 h were approximately 26, 24, and 23 μm, respectively (Figure 8). The overall difference of approximately 3 μm was comparable to the dimensions of individual deposited particulate features (approximately 2–3 μm), suggesting broadly similar coating formation during extended spraying. The measured viscosities of the 7.4 wt.% slurry before and after the experiment were 13.49 and 13.75 cP, respectively (Table 4). These results indicate that the selected slurry maintained practical sprayability and continued to form porous coatings over the 8 h period.

**Figure 9:**
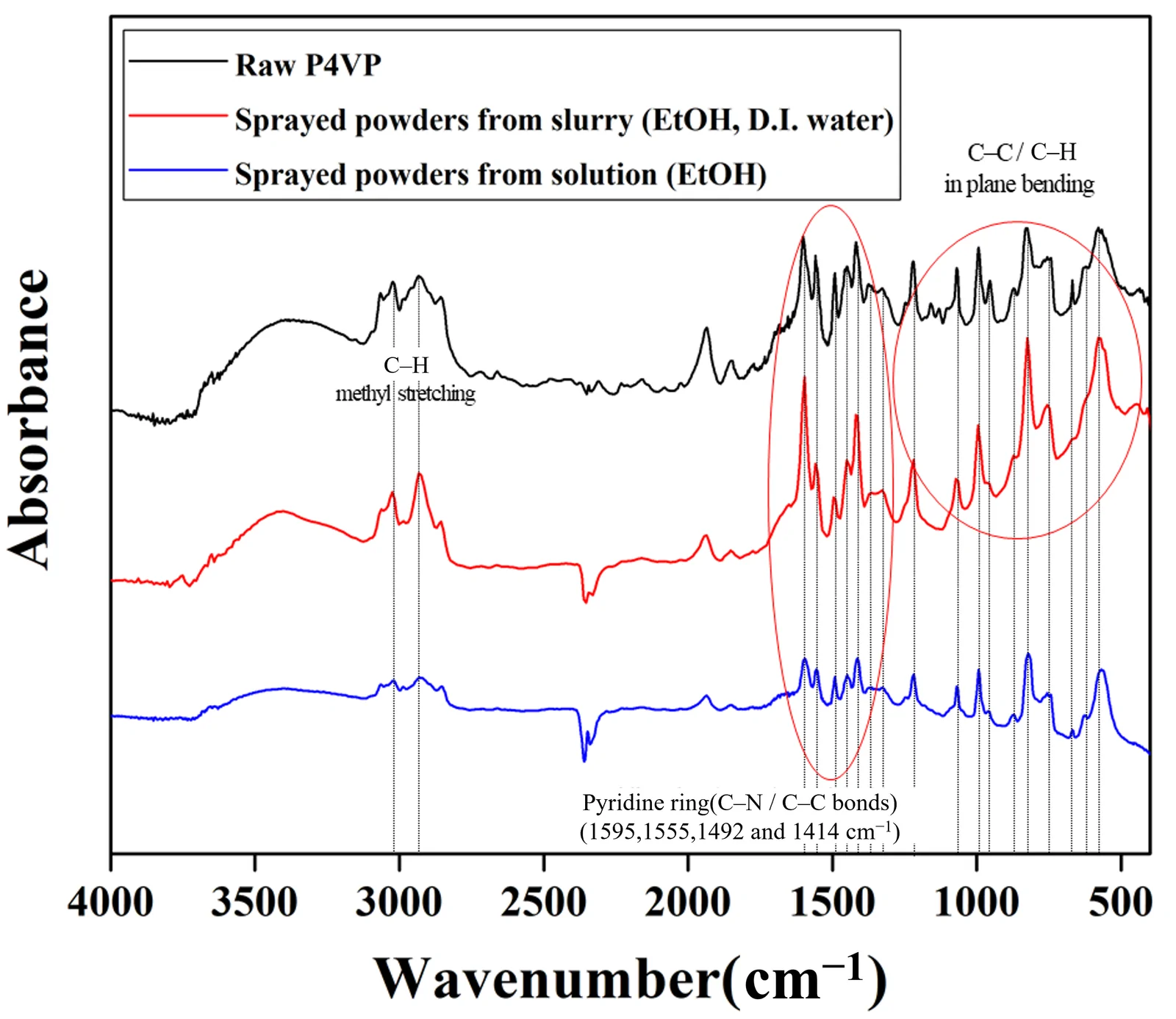
FTIR spectra of untreated P4VP and material recovered after ESSD from solution and composite-slurry precursors. The red ellipses indicate characteristic absorption regions highlighted for comparison among the samples.

Figure 9 compares FTIR spectra of untreated P4VP and materials recovered after ESSD from solution and composite-slurry precursors. The characteristic pyridine-ring absorption bands in the 1500–1600 cm^−1^ region were retained across the samples, indicating that the principal P4VP functionality was preserved during precursor preparation and deposition. This result supports the suitability of the prepared formulations for producing P4VP-containing coatings by ESSD.

**Figure 10:**
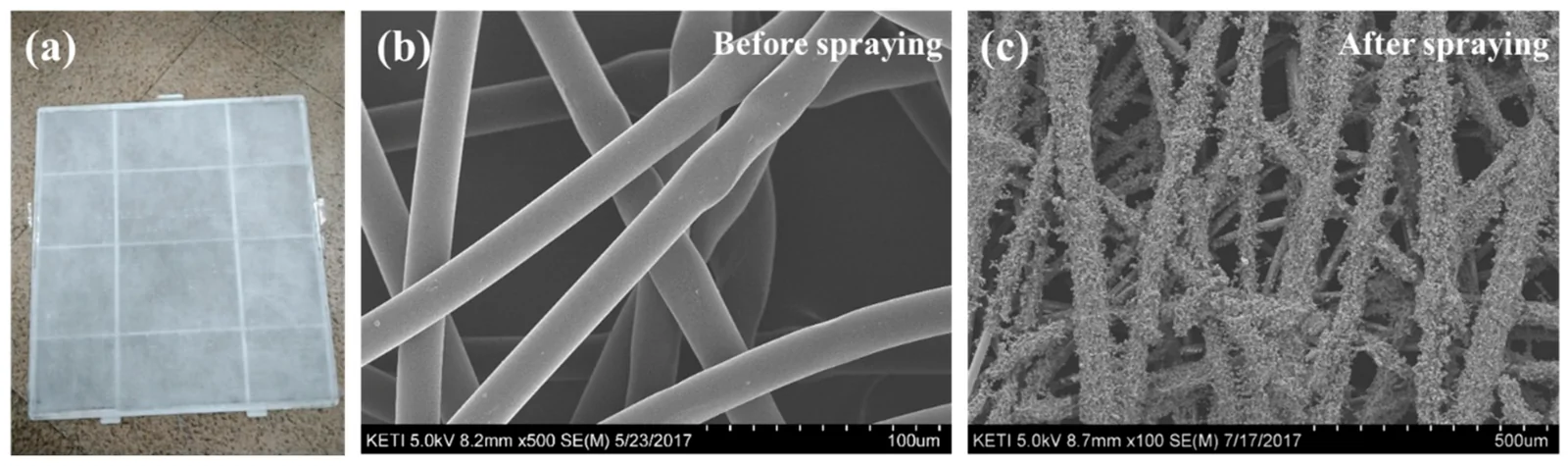
(**a**) Photograph of the uncoated air-purifier filter scaffold and FE-SEM images of the fibrous surface (**b**) before and (**c**) after P4VP coating deposition by ESSD.

To evaluate the applicability of ESSD to a practical filter substrate, the same 7.4 wt.% P4VP composite slurry was deposited onto a commercial air-purifier filter scaffold. The operating conditions were adjusted to accommodate the larger coating area and three-dimensional fibrous geometry, as described in Section 2.3. Figure 10a shows the uncoated filter, while Figure 10b and Figure 10c compare the fiber surfaces before and after deposition, respectively. FE-SEM observations show a porous particulate P4VP coating on the fibers, demonstrating that the composite-slurry ESSD process can be applied to a practical filter scaffold.

**Figure 11:**
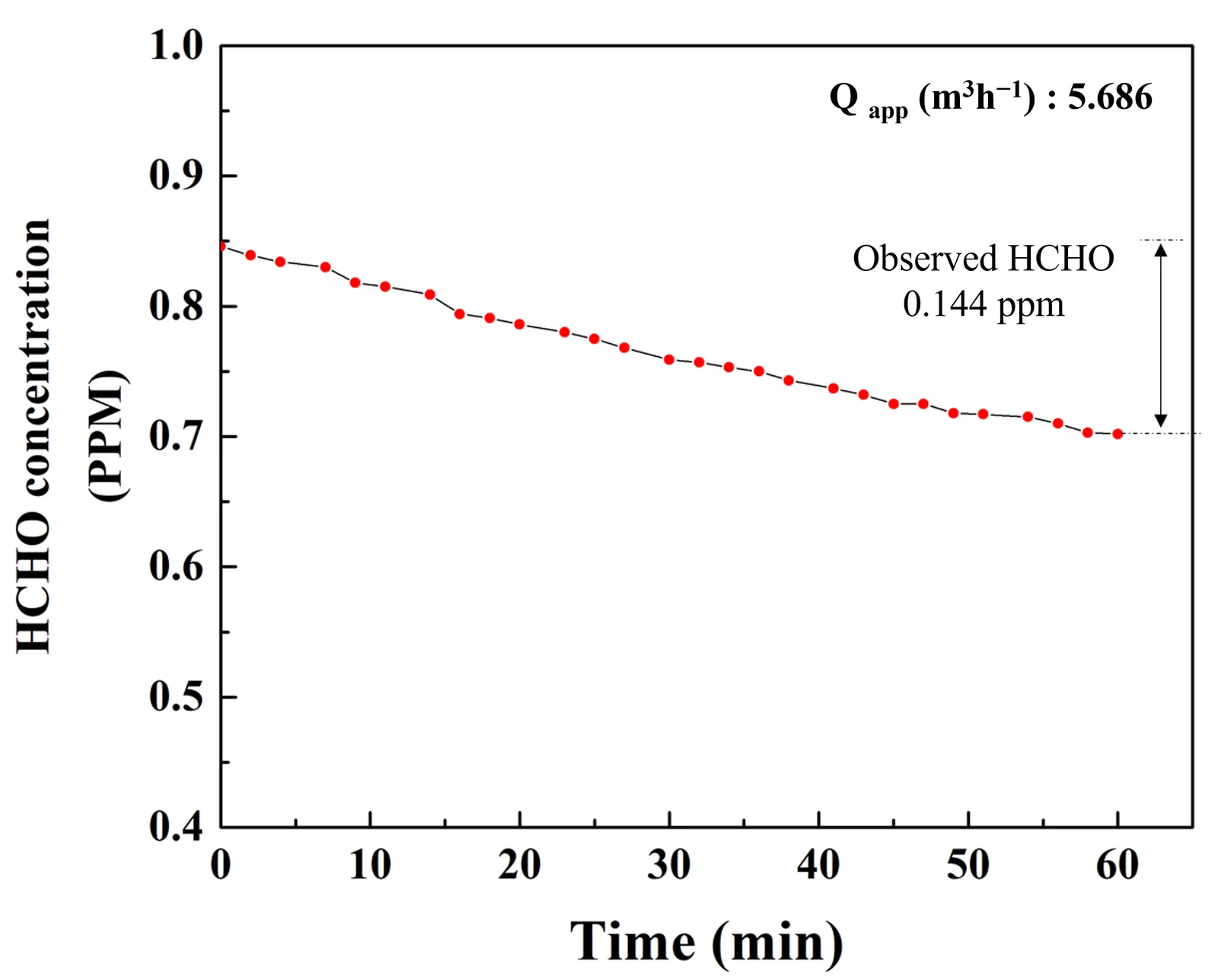
HCHO concentration–time profile recorded during the preliminary 60 min chamber assessment of an air purifier equipped with a P4VP-coated filter (nominal chamber volume: 30 m^3^). The 28 observations are provided in Supplementary Table S1.

As shown in Figure 11, the measured HCHO concentration decreased from 0.846 to 0.702 ppm in 60 min, corresponding to a reduction of 0.144 ppm. The 28 observations in Supplementary Table S1 were fitted by ordinary least-squares regression of ln C(t) against elapsed time in hours using ln C(t) = a − kt, with a freely fitted intercept. The resulting rate constant was *k* = 0.1895 h^−1^ (R^2^ = 0.9922), with a regression standard error of 0.0033 h^−1^ and a nominal 95% confidence interval of 0.1828–0.1963 h^−1^. The fitted initial concentration was 0.8410 ppm. The apparent concentration-decay-based air-cleaning rate (*Q_app_*) was calculated using Equation (2):

(2) *Q_app_* = *V* × *k* = 30 m^3^ × 0.1895289 h^−1^ = 5.686 m^3^ h^−1^

where *Q_app_* is the apparent concentration-decay-based air-cleaning rate (m^3^ h^−1^), *V* is the nominal chamber volume (30 m^3^), and k is the fitted decay constant (h^−1^). The fitted value gives *Q_app_* ≈ 5.69 m^3^ h^−1^. This value describes the concentration decay measured during operation of the complete air-purifier system equipped with the coated filter. As matched background-decay and uncoated-filter controls were not available, it is reported as an uncorrected, system-level apparent rate rather than a background-corrected CADR or a quantitative measure of the P4VP coating alone.

## 4. Conclusions

This study demonstrated the fabrication of porous P4VP coatings by ESSD using ethanol–water composite slurries prepared through controlled nonsolvent addition. Under the investigated conditions, composite-slurry deposition produced a more spatially consistent porous particulate layer on silicon wafers than the solution precursor, and the same processing approach was successfully extended to a three-dimensional fibrous filter scaffold. The observed particle morphologies are consistent with a possible evaporation-driven pathway discussed in the droplet-drying and electrospraying literature [35,36,37], providing a basis for further studies of particle structure and coating architecture.

The selected 7.4 wt.% slurry exhibited a principal volume-weighted DLS peak at 13.78 nm, and characteristic pyridine functionality was retained following deposition. The 8 h spraying experiment showed continued formation of porous coatings with comparable thicknesses. In a preliminary 30 m^3^ chamber assessment, the HCHO concentration decreased from 0.846 to 0.702 ppm within 1 h, corresponding to an apparent concentration-decay-based air-cleaning rate of approximately 5.69 m^3^ h^−1^. This system-level observation demonstrates the feasibility of evaluating the coated filter in an air-purifier assembly, while controlled tests are needed to quantify the contribution of the P4VP coating itself. Overall, composite-slurry ESSD provides a promising route to the preparation and morphological control of porous polymer coatings on planar and fibrous substrates.

## Figures and Tables

**Table 1:** Physicochemical properties, P4VP solubility, and spray stability of 34 solvents.

Solvent	Chemical Formula	Dielectric Constant (κ) [26]	Density, ρ (g cm^−3^) [27]	Surface Tension, γ (N m^−1^) [28]	Viscosity, μ (Pa s) [27]	P4VP Solubility	Spray Stability
Formic acid	CH_2_O_2_	51.1	1.220 †	3.71× 10-2 †	1.57× 10^-3^	Soluble	Stable
Acetone	C_3_H_6_O	20.7	0.784	2.35× 10^-2^	3.06× 10^-4^	Soluble	Stable
Ethanol	C_2_H_6_O	24.3	0.785	2.20× 10^-2^	1.07× 10^-3^	Soluble	Stable
Tetrahydrofuran	C_4_H_8_O	7.58	0.889 †	2.64× 10^-2^	4.56× 10^-4^	Soluble	Stable
Tri-ethylene glycol	C_6_H_14_O_4_	23.7 †	1.126 †	4.52× 10^-2^ [29]	4.90× 10^-2^ † [29]	Soluble	Stable
Dimethyl formamide	C_3_H_7_NO	36.7	0.944	3.52× 10^-2^	8.02× 10^-4^	Soluble	Stable
Water	H_2_O	78.4	0.997	7.20× 10^-2^	8.90× 10^-4^	Insoluble	Unstable
Isopropyl alcohol	C_3_H_8_O	19.9	0.781	2.09× 10^-2^	2.04× 10^-3^	Soluble	Stable
Propylene carbonate	C_4_H_6_O_3_	64.9	1.200	4.11× 10^-2^	2.53× 10^-3^	Soluble	Stable
N-Methyl-2-pyrrolidone	C_5_H_9_NO	32.2	1.028	4.08× 10^-2^	1.67× 10^-3^	Soluble	Stable
n-Propanol	C_3_H_8_O	20.1	0.800	2.33× 10^-2^	1.96× 10^-3^	Soluble	Stable
n-Butanol	C_4_H_10_O	17.5	0.806	2.49× 10^-2^	2.54× 10^-3^	Soluble	Stable
Acetic acid	C_2_H_4_O_2_	6.2 †	1.044	2.71× 10^-2^	1.06× 10^-3^	Insoluble	Stable
Methanol	CH_4_O	32.7	0.787	2.21× 10^-2^	5.44× 10^-4^	Soluble	Stable
Ethyl acetate	C_4_H_8_O_2_	6.02	0.895	2.34× 10^-2^	4.23× 10^-4^	Insoluble	Stable
Acetonitrile	C_2_H_3_N	37.5 †	0.777	2.87× 10^-2^	3.43× 10^-4^	Soluble	Stable
Chloroform	CHCl_3_	4.81 †	1.480	2.67× 10^-2^	5.37× 10-4	Insoluble	Stable
Dichloromethane	CH_2_Cl_2_	8.93	1.317	2.78× 10^-2^	4.13× 10-4	Soluble	Stable
1,2-Dichloroethane	C_2_H_4_Cl_2_	10.4	1.246	3.19× 10^-2^	7.79× 10-4	Soluble	Stable
1,4-Dioxane	C_4_H_8_O_2_	2.21	1.028	3.28× 10^-2^	1.18× 10-3	Soluble	Stable
Anisole	C_7_H_8_O	4.33	0.994	3.51× 10^-2^	1.06× 10-3	Insoluble	Stable
Diethyl ether	C_4_H_10_O	4.27 †	0.708	1.67× 10^-2^	2.24× 10-4	Insoluble	Stable
Dimethyl carbonate	C_3_H_6_O_3_	3.1	1.063	2.85× 10^-2^	5.85× 10-4	Insoluble	Unstable
Dimethyl sulfoxide	C_2_H_6_OS	46.7	1.095	4.35× 10^-2^	1.99× 10-3	Insoluble	Unstable
Pentane	C_5_H_12_	1.84 †	0.621	1.55× 10^-2^	2.24× 10-4	Insoluble	Unstable
Hexane	C_6_H_14_	1.88 †	0.655	1.79× 10^-2^	3.00× 10-4	Insoluble	Unstable
Heptane	C_7_H_16_	1.92 †	0.680	1.97× 10^-2^	3.86× 10-4	Insoluble	Unstable
Cyclopentane	C_5_H_10_	1.97 †	0.740	2.19× 10^-2^	4.16× 10-4	Insoluble	Unstable
Cyclohexane	C_6_H_12_	2.02 †	0.774	2.47× 10^-2^	8.94× 10-4	Insoluble	Unstable
Benzene	C_6_H_6_	2.27	0.874	2.82× 10^-2^	6.04× 10-4	Insoluble	Unstable
Xylene (m-xylene)	C_8_H_10_	2.37 †	0.864 †	2.89× 10^-2^ †	6.20× 10-4†	Insoluble	Unstable
Toluene	C_7_H_8_	2.38	0.862	2.79× 10^-2^	5.60× 10^-4^	Insoluble	Unstable
Ethyl methyl carbonate	C_4_H_8_O_3_	2.96 [30]	1.006 [31]	–	6.50× 10^-4^ [30]	Insoluble	Unstable
Dibutyl ether	C_8_H_18_O	3.08	0.764	2.31× 10^-2^ †	6.37× 10-4	Insoluble	Unstable

Note: † indicates literature values reported at 20 °C; other literature values are primarily reported at 25 °C.

**Table 2:** Spray-footprint diameters of selected pure solvents under preliminary spraying conditions.

Solvent	Spray Condition		Deposition Diameter
	Flowrate	Nozzle to Substrate Distance	
Acetic acid	3 mL/hr	12 cm	3.5 cm
Ethyl acetate	3 mL/hr	12 cm	2.9 cm
Chloroform	3 mL/hr	12 cm	3.0 cm
Anisole	3 mL/hr	12 cm	3.2 cm
Diethyl ether	3 mL/hr	12 cm	2.6 cm

**Table 3:** P4VP precursor formulations and ESSD operating conditions.

P4VP Precursor			Fabrication Condition		Spray Condition	
			Solvent	P4VP	Flowrate	Nozzle to Substrate Distance
	Solution		EtOH: 10 mL	0.1 g	3 mL/hr	12 cm
Slurry (composite solvent)	S1	12.3 wt.%	EtOH: 28 mL D.I. water: 28 mL	7.0 g	3 mL/hr	12 cm
	S2	9.1 wt.%	EtOH: 28 mL D.I. water: 28 mL	5.0 g	3 mL/hr	12 cm
	S3	8.2 wt.%	EtOH: 28 mL D.I. water: 28 mL	4.5 g	3 mL/hr	12 cm
	S4	7.4 wt.%	EtOH: 28 mL D.I. water: 28 mL	4.0 g	3 mL/hr	12 cm
	S5	4.8 wt.%	EtOH: 28 mL D.I. water: 28 mL	2.5 g	3 mL/hr	12 cm
	S6	2.0 wt.%	EtOH: 28 mL D.I. water: 28 mL	1.0 g	3 mL/hr	12 cm
	S7	1.0 wt.%	EtOH: 28 mL D.I. water: 28 mL	0.5 g	3 mL/hr	12 cm

**Table 4:** Dynamic viscosities of P4VP composite slurries determined from Ubbelohde capillary measurements and composition-estimated densities.

P4VP Precursor		Viscosity, η (cP)
S1	12.3 wt.%	39.12
S2	9.1 wt.%	25.72
S3	8.2 wt.%	18.40
S4	7.4 wt.%, (before 8 h spraying)	13.49
S4	7.4 wt.%, (after 8 h spraying)	13.75

## Data Availability

The original contributions presented in this study are included in the article/Supplementary Material. Further inquiries can be directed to the corresponding author.

## Supplementary Materials

The following supporting information can be downloaded at: https://www.mdpi.com/article/10.3390/ma19214744/s1, Table S1. Time-dependent HCHO concentrations measured during the preliminary 60 min chamber assessment (nominal volume: 30 m^3^). The table contains the 28 observations used for the log-linear fit.
