# Supplementary material for: Porous Poly(4-vinylpyridine) Coatings Prepared by Electrostatic Slurry Spray Deposition: Morphological Control and Preliminary Formaldehyde-Reduction Assessment
Source: Materials (Basel). 2026 Nov 8;19(21):4744. doi: 10.3390/ma19214744 (PMC13764831; doi:10.3390/ma19214744)
Supplement: Supplementary file 1 [file materials-19-04744-s001.zip › materials-4633065-supplementary.pdf]

Supplementary Table S1

Time-dependent HCHO concentrations measured during the preliminary 60 min chamber assessment (nominal volume: 30 m^3). The table contains the 28 observations used for the log-linear fit.

Time (min)	HCHO (ppm)	Time (min)	HCHO (ppm)
0	0.846	32	0.757
2	0.839	34	0.753
4	0.834	36	0.750
7	0.830	38	0.743
9	0.818	41	0.737
11	0.815	43	0.732
14	0.809	45	0.725
16	0.794	47	0.725
18	0.791	49	0.718
20	0.786	51	0.717
23	0.780	54	0.715
25	0.775	56	0.710
27	0.768	58	0.703
30	0.759	60	0.702

Note: This table reports observed concentrations only; the apparent air-cleaning rate reported in the manuscript is not background-corrected and does not isolate the P4VP-specific contribution.
